# Granulovacuolar Degeneration in Hippocampus of Neurodegenerative Diseases: Quantitative Study

**DOI:** 10.1155/2016/6163186

**Published:** 2016-10-23

**Authors:** Maher Kurdi, Esther Chin, Lee Cyn Ang

**Affiliations:** ^1^Department of Pathology, London Health Science Centre, Western University, London, ON, Canada N6A 5C1; ^2^Department of Pathology, King Abdul-Aziz University, Jeddah, Saudi Arabia; ^3^Department of Pathology, Schulich School of Medicine & Dentistry, Western University, London, ON, Canada N6A 5C1

## Abstract

*Background*. Granulovacuolar degeneration (GVD) is one of the pathological features long associated with Alzheimer's disease (AD) and normal aging.* Aim*. We investigate the frequency of GVDs in AD, other neurodegenerative diseases, and normal aging, with attempt to determine whether the GVD has preponderance in any particular neurodegenerative disease other than AD.* Materials and Methods*. A retrospective review of 111 autopsied cases with a variety of neurodegenerative diseases and 70 control cases without pathological evidence of neurodegeneration was evaluated quantitatively. The microscopic examination was applied on coronal sections of hippocampi using Hematoxylin and Eosin (H&E) and Bielschowsky silver impregnation. The mean percentage of neurons with GVDs was calculated through all sectors of Ammon's horn for each case.* Result*. We found that neurons with GVD, in cases with or without neurodegenerative diseases, were found predominantly in CA1 and CA2 sectors of hippocampus. The GVD count in AD was significantly increased in CA1 and CA2 compared to other neurodegenerative cases as well as normal aging controls. In AD/LBD there was a significant increase in GVD in CA1 whereas in LBD there was no significant change in GVD.* Conclusions.* The frequency of GVD in AD is due to the disease process and attributes the increase for AD/LBD to the AD component.

## 1. Introduction

GVD was first reported by Simchowicz in the hippocampus in cases of senile dementia in 1911 [1] and its ultrastructural characteristics were subsequently described by Hirano et al. in 1968 [2]. Microscopically, it is depicted by intraneuronal accumulation of large membrane-bound vacuoles harboring argyrophilic granules [3]. Ultrastructurally, the cytoplasmic vacuoles are 3–5 *μ*m in diameter, each containing a single granule measuring 0.5–1.5 *μ*m wide [4]. These granules contain epitopes for a group of protein constituents linking GVD with neurodegenerative processes. The protein constituents described in GVD, such as neurofilament protein, phosphorylated tau, ubiquitin, tropomyosin, vimentin, and, interestingly, mitotic proteins, are also represented in the autophagic ubiquitin proteasome system [5]. Therefore, GVD is postulated to be derived from cellular autophagic processes and appears when phosphorylated tau begins to aggregate into neurofibrillary tangles [6]. Nevertheless, GVD was not considered to be directly involved in neurofibrillary tangle (NFT) formation or A*β* plaque deposition [2]. Studies have examined the prevalence and distribution of GVD in human postmortem brains [7] as well as rat brains [8]. Tomlinson and Kitchener [9] have measured the degree of GVD in both normal aging population and demented patients. Generally, pyramidal neurons in the subiculum and CA1 zone of the hippocampus are most susceptible to the GVD [10]. Ventrolateral quadrant of the hippocampus is more vulnerable to GVD than the rest of the hippocampus while parahippocampal cortex is much less vulnerable [11]. Neurons in CA2, CA3, CA4, and subiculum are less frequently affected by GVD [12]. Rare GVDs were described in neocortex, hypothalamus, amygdala, and the paramedian nuclei of the midbrain [13]. The present study attempts to establish statistically the significance of GVD in the hippocampus in different neurodegenerative diseases (including AD, LBD, and AD with LBD, AGD, CBD, FTLD-U, MSA, PD, PiD, and PSP) in comparison to normal aging, in order to determine whether the degree of GVD is due to disease or aging process.

## 2. Materials and Methods

### 2.1. Specimens

We retrospectively examined 181 postmortem brains with ages ranging from 43 to 95 years in the period between 1993 and 2005 at the University Hospital (London Health Sciences Center), London, Ontario, Canada. Of these, 70 were control cases with no evidence of clinical dementia or movement disorders, and disclosed only age-related changes without neurodegenerative findings on neuropathological examination. The brains of neurodegenerative cases (*n* = 111) were examined as well. These included 56 AD, 9 AD/LBD, 2 AGD, 4 CBD, 10 FTLD-U, 10 LBD, 5 MSA, 3 PD, 2 PiD, and 10 PSP. These cases showed clinically progressive dementia or movement disorders and exhibited definite neuropathological findings of the respective diseases as determined by our neuropathologists. Immunohistochemistry for tau (PHF), alpha-synuclein, beta-amyloid, and ubiquitin has been applied on sections of the neurodegenerative diseases and when necessary to the control cases. The Bielschowsky silver impregnation is performed on all hippocampal sections and frontal sections. The AD cases were diagnosed using the NFT staging (Braak and Braak) and plaque score (CERAD). The pathological diagnosis of AD was based on NFT staging of 4 or above and plaque score of 2 or above. Coronal sections of the hippocampus at the level of lateral geniculate body were taken for examination. We reviewed the age, gender, clinical history, and diagnosis from medical records of all cases. This study has been approved by Research Ethics Board, University of Western Ontario, and LHSC in London, Canada.

### 2.2. Tissue Preparation and Histochemistry

**Figure 1:**
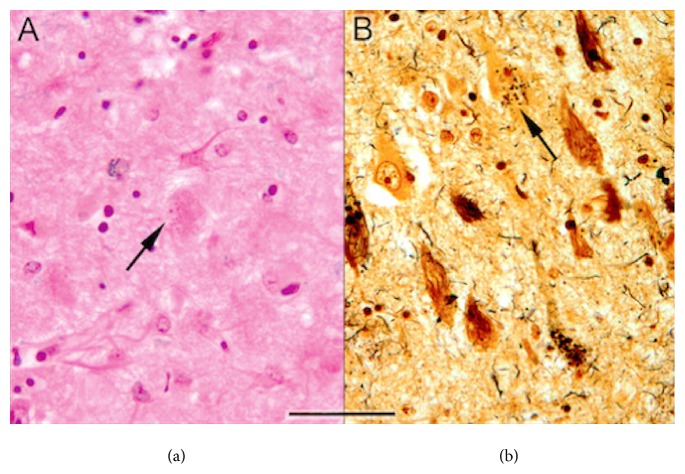
(a) Sections treated with H&E (×25). It shows granulovacuolar degeneration; (b) section treated with Bielschowsky silver stain (×25). It shows granulovacuolar granules.

All specimens were fixed in 20% neutral buffered formalin and embedded in paraffin wax. Sections were stained with both Hematoxylin and Eosin (H&E) and Bielschowsky silver impregnation and examined under light microscopy (Figures 1(a) and 1(b)).

### 2.3. Quantitative Analysis of GVDs

The area analyzed included all four sectors of hippocampus (Ammon's horn). The total numbers of the neurons and the number of neurons affected by GVD were counted sequentially over the entire area with aid of Bielschowsky stain, using light microscopy at magnification of 250 times. The total average mean value for each disease in each sector was calculated and regarded as a mean percentage of GVD:

(1) Mean  Percentage  of  GVD=Number  of  neurons  affected  by  GVDTotal  numbers  of  neurons  in  the  entire  area×100.

Comparison of means between disease groups as well as with the controls and the test significance were determined using two tailed statistical methods, one-way ANOVA and Post Hoc Scheffe test. The proportions were transformed using the arcsin (sqrt) to improve the equity of variances and the normal distribution. The one-way ANOVA is used to compare the effect of the disease on mean percentage of GVDs in each hippocampus. The Post Hoc Scheffe test is used to compare the mean percentage of GVD of disease groups and controls to specify which disease group has the significant effect. Because ANOVA approach was not available for nonparametric comparisons, Dunn's test is used to overcorrect the differences. Dunn's test is a generalization of the Bonferroni correction and is then overly conservative.

## 3. Results

On nonparametric analysis of our data with respect to age and sex, we found that 37 of controls cases were females (52.9%) while 33 of them were males (47.1%). The cases with neurodegenerative diseases were 61 females (58.7%) and 43 males (41.4%). Overall, there were 98 females (56.3%) and 76 males (41.4%) of both control and neurodegenerative cases.

Statistical analysis for control and neurodegenerative cases for age and sex is summarized in Table 1.

**Figure 2:**
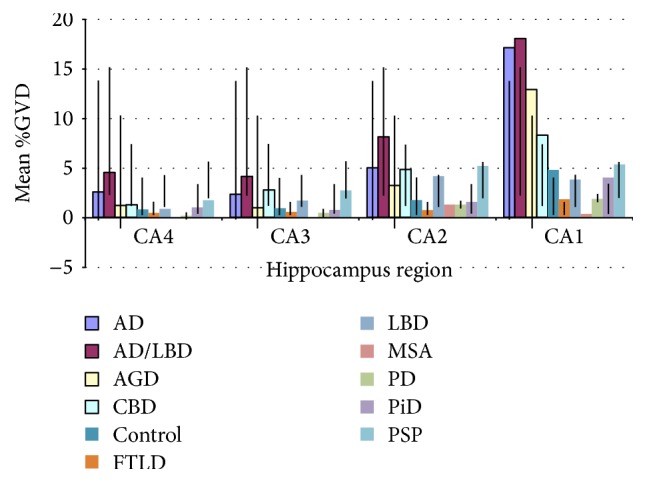
The figure outlines the main findings of the observational and statistical results with addressed errors bars marking standard deviation (SD).

*Note*. Tables 2, 3, 4(a), and 4(b), and Figure 2 outline the main findings of the following observational and statistical results.

### 3.1. Observational Results

#### 3.1.1. GVD in Control and Neurodegenerative Patients

Qualitatively, GVD is present in CA1 in all AD, AD/LBD, AGD, CDB, LBD, PSP, and PD, taking into account that the number of PD and AGD cases studied is very small (3 and 2 cases, resp.). In the control cases and other neurodegenerative diseases (FTLD, MSA, and PiD), CA1 is the most affected sector by GVD. GVD in CA1 was found in 62 of the 70 control cases. GVD was not found in 8 cases with age below 60 years in CA1 sector. Qualitatively, neurons with GVD in CA1 are apparently found in all normal controls with the age above 60 years. The CA2 is the second most likely affected sector by GVD, followed by CA4 with the least frequency in CA3 (see Table 2).

Based on the qualitative presence or absence of GVD in the different sectors of the hippocampi, we are unable to assess the pathological relevance of GVD in the different neurodegenerative diseases and nondegenerative cases above 60 years.

### 3.2. Statistical Results

The means and standard deviations for the individuals with neurodegenerative diseases and controls are in Table 3.

#### 3.2.1. GVD at CA1 Sector in Neurodegenerative and Control Cases (Table 4(a))

AD (*p* < 0.0001) and AD/LBD (*p* = 0.0118, <0.05) differed significantly in mean percentage of neurons with GVD in CA1 from the normal controls. LBD (without the AD component) shows no significant increase in neurons with GVD from control. Similarly, the other neurodegenerative diseases showed no significant increase in neurons with GVD compared to normal controls. Although in AGD, CDB, and PSP the means are higher than the normal controls but the differences are not statistically significant. The number of cases for AGD and CBD are small, and therefore the results are not considered meaningful.

The mean GVD counts of AD are also statistically significant higher than FTLD-U, LBD, PSP, and MSA. Applying Dunn's test, the only significant difference occurs between AD and FTLD-U and MSA. Similarly the mean GVD count of AD/LBD is significantly higher than FTLD-U, and MSA and Dunn's test are also significant.

#### 3.2.2. GVD at CA2 Sector in Neurodegenerative and Control Cases (Table 4(b))

In CA2 sector, only AD (*p* = 0.0287, <0.05) and AD/LBD (*p* = 0.049, <0.05) show significant difference in mean percentage of neurons with GVD from the control. Although, in AGD, CDB, LBD, and PSP, the means are higher than the controls, the differences are not statistically significant. The mean percentage of neurons with GVD is not significantly different in AD compared to any of the other neurodegenerative diseases.

#### 3.2.3. GVD at CA3 Sector in Neurodegenerative and Control Cases

Although the individual mean values of GVD counts for AD, AD/LBD, AGD, CDB, LBD, and PSP are higher than the control, there is no significant difference in mean percentage of neurons with GVD between neurodegenerative diseases (including AD) and control cases. There are also no significant differences in mean percentage of neurons with GVD between AD and any other neurodegenerative diseases.

#### 3.2.4. GVD at CA4 Sector in Neurodegenerative and Control Cases

Although the individual mean values of GVD counts for AD, AD/LBD, AGD, CDB, LBD, PiD, and PSP are higher than the control, only AD (*p* = 0.0283, <0.05) shows a significant difference in the mean percentage of neurons of GVD compared to control cases. There is no significant difference in mean percentage of neurons with GVD between AD and other neurodegenerative diseases.

## 4. Discussion

Although GVD of hippocampal pyramidal neurons is usually associated with senile dementia of the Alzheimer's disease, it has been found in normal aging brain [10], as well as brains from patients with PSP, PiD, PD, Guam's Parkinson-ALS-dementia diseases [2], and Down's syndrome [14]. The degree of GVD per neuron is also found to correlate with the severity of dementia [15].

GVD can be detected with routine histological stains such as Hematoxylin and Eosin, silver impregnation techniques (such as Bielschowsky), and other immunohistochemical stains including tau [16], TDP-43 [17], ubiquitin [18], GSK [16], CHMP2B [19], and Casein-kinase 1 (CK-1) [20].

Our data show that GVD can be identified qualitatively in neurons of the hippocampi of patients with the neurodegenerative diseases as well as cognitively normal controls above the age of 60 years. These findings replicated these of Xu et al. [7] who found 54 of 75 nondemented cases above 60 years harboring GVD in CA1 and CA2, while one case from the 21 patients below age of 60 showed GVD. Tomlinson and Kitchener [9] measured the degree of GVD in both mentally normal aging population and dementia patients. In their study, the topographic distribution of GVD within the hippocampus was found to be identical in 30 nondemented elderly people and 25 people with senile dementia. Only two examined cases below age of 50 showed neuronal cells affected with GVD. Is et al. [8] examined 44 experimental rats between age of one month and 24 months and they found that GVD is present in all areas of the hippocampus in rats with increasing the age.

In our study, the quantitative data on the percentages of hippocampal neurons with GVD among neurodegenerative cases and cognitively normal controls can help in distinguishing the neurodegenerative effect from age-related changes. Among the neurodegenerative diseases, only AD and AD/LBD showed statistically significant increase in percentages of neurons with GVD compared with age matched normal controls in CA1 and CA2. Interestingly, LBD without any AD component has no effect on percentages of neurons with GVD in CA1 and CA2. In CA3, there is no significant difference in percentage of neurons with GVD between any neurodegenerative diseases and the aged match controls. In CA4, only AD showed an impact of increasing the percentages of neurons with GVD.

Xu et al. [7] identified GVDs in 43 dementia cases, particularly the AD cases, and they found that the difference between the number of affected neurons with GVD in AD and in non-AD dementia was statistically significant. In their study, they included AD, Pick's disease (PiD), multi-infarct dementia, and atypical dementia (defined as non-Alzheimer's, non-Pick's dementia with Fahr's syndrome), whereas our study is the first to examine quantitatively the presence of hippocampal GVD in AD/LBD, AGD, CDB, LBD, PSP, and PD in addition to AD, PiD, and normal aging.

## 5. Conclusions 

GVD can be identified in nondemented patients linked to aging process or non-Alzheimer's neurodegenerative diseases. The increased frequency of GVD in hippocampus predominantly at the CA1 and CA2 in AD and AD/LBD suggests that the AD disease process does enhance the generation of GVD. Our finding further supports that LBD without the AD component has no effect on the frequency of GVD in the hippocampus. We propose that quantifying GVD in CA1/CA2 on a single standard section of the hippocampus with the Bielschowsky stain could be a useful diagnostic marker for AD, especially in institutions where extensive sampling of brains and the use of a large panel of immunostain are not feasible.

## Figures and Tables

**Table 1:** The table shows the mean age and sex ration for all neurodegenerative and control cases.

	Age			Sex			
				Female		Male	
Cases	*n*	Mean	SD	*n*	%	*n*	%
AD	56	77.75	10.78	40	70.18	17	29.82
AD/LBD	9	80.62	7.28	5	55.56	4	44.44
AGD	2	82.50	4.94	0	0	2	100
CBD	4	75.75	6.13	2	50	2	50
Control	70	73.57	8.19	37	52.86	33	47.14
FTLD	10	72.16	13.49	4	66.67	2	33.33
LBD	10	77.40	8.32	5	50	5	50
MSA	5	64.20	12.27	2	60	3	60
PD	3	75.33	5.77	1	66.67	2	66.67
PSP	10	73.66	5.60	1	83.33	5	83.33
PiD	2	70.50	0.70	1	50	1	50

Cases	181	75.32	9.54	98	56.32	76	43.68

**Table 2:** The table shows granulovacuolar degeneration in control and neurodegenerative patients. The CA2 is the second most likely affected sector by GVD, followed by CA4 with the least frequency in CA3.

Cases	*n*	CA1	CA2	CA3	CA4
AD	56	56	46	32	42
AD/LBD	9	9	9	7	7
AGD	2	2	2	1	2
CBD	4	4	3	2	2
Control	70	62	41	26	20
FTLD-U	10	8	5	4	4
LBD	10	10	8	4	5
MSA	5	3	4	0	1
PD	3	3	2	2	1
PiD	2	1	1	1	1
PSP	10	10	6	5	5

**Table 3:** The table shows the means and standard deviations for the individuals with neurodegenerative diseases and controls.

		CA4		CA3		CA2		CA1	
Cases	*n*	Mean	SD	Mean	SD	Mean	SD	Mean	SD
AD	56	2.6	5.1637	2.356	4.5980	4.945	5.7498	17.089	10.9065
AD/LBD	9	4.503	6.4548	4.145	5.5396	8.084	6.0677	18.013	9.8355
AGD	2	1.196	0.1701	0.950	1.3443	3.207	1.2077	12.937	6.6612
CBD	4	1.226	1.6608	2.692	3.2699	4.853	4.8485	8.34	4.5758
Control	70	0.865	2.7350	0.949	4.0331	1.827	2.9555	4.832	6.3212
FTLD-U	10	0.445	0.6629	0.522	0.7976	0.797	1.0281	1.865	1.9706
LBD	10	0.883	1.2036	1.759	2.6480	4.206	4.3640	3.788	3.8121
MSA	5	0.117	0.2631	0	0.0000	1.339	0.9887	0.412	0.8307
PD	3	0.179	0.3104	0.427	0.4081	1.333	1.8074	1.956	0.1378
PiD	2	1.026	1.4505	0.819	1.1592	1.562	2.2097	4.062	5.7452
PSP	10	1.715	2.8028	2.735	5.0261	5.194	6.0062	5.401	7.5781

**Table:** (a) GVD at CA1 sector in neurodegenerative and control cases. Dependent Variable: t_CA1. For AD *p* < 0.0001 and AD/LBD *p* = 0.0118, <0.05

Cases	AD	AD/LBD	AGD	CBD	Control	FTLD	LBD	MSA	PD	PSP	PiD
AD		1.0000	1.0000	0.9886	<0.0001	0.0002		0.0017	0.4712	0.0401	0.7808
AD/LBD	1.0000		1.0000	0.9889	0.0118	0.0161	0.1775	0.0141	0.5533	0.2373	0.7948
AGD	1.0000	1.0000		1.0000	0.9727	0.8754	0.9843	0.6968	0.9830	0.9901	0.9928
CBD	0.9886	0.9889	1.0000		0.9945	0.9318	0.9982	0.7644	0.9978	0.9993	0.9994
Control	<0.0001	0.0118	0.9727	0.9945		0.9969	1.0000	0.9306	1.0000	1.0000	1.0000
FTLD	0.0002	0.0161	0.8754	0.9318	0.9969		0.9997	0.9999	1.0000	0.9990	1.0000
LBD	0.0223	0.1775	0.9843	0.9982	1.0000	0.9997		0.9792	1.0000	1.0000	1.0000
MSA	0.0017	0.0141	0.6968	0.7644	0.9306	0.9999	0.9792		0.9999	0.9639	0.9999
PD	0.4712	0.5533	0.9830	0.9978	1.0000	1.0000	1.0000	0.9999		1.0000	1.0000
PSP	0.0401	0.2373	0.9901	0.9993	1.0000	0.9990	1.0000	0.9639	1.0000		1.0000
PiD	0.7808	0.7948	0.9928	0.9994	1.0000	1.0000	1.0000	0.9999	1.0000	1.0000	

**Table:** (b) GVD at CA2 sector in neurodegenerative and control cases. Dependent Variable: t_CA2. For AD *p* = 0.0287, <0.05 and AD/LBD *p* = 0.049, <0.05

Cases	AD	AD/LBD	AGD	CBD	Control	FTLD	LBD	MSA	PD	PSP	PiD
AD		0.9438	1.0000	1.0000	0.0287	0.4569	1.0000	0.9911	0.9955	1.0000	0.9992
AD/LBD	0.9438		0.9998	0.9989	0.0499	0.1311	0.9629	0.7454	0.8478	0.9645	0.9455
AGD	1.0000	0.9998		1.0000	0.9997	0.9981	1.0000	1.0000	1.0000	1.0000	1.0000
CBD	1.0000	0.9989	1.0000		0.9910	0.9737	1.0000	0.9997	0.9996	1.0000	0.9999
Control	0.0287	0.0499	0.9997	0.9910		1.0000	0.9487	1.0000	1.0000	0.9456	1.0000
FTLD	0.4569	0.1311	0.9981	0.9737	1.0000		0.9288	1.0000	1.0000	0.9259	1.0000
LBD	1.0000	0.9629	1.0000	1.0000	0.9487	0.9288		0.9997	0.9997	1.0000	0.9999
MSA	0.9911	0.7454	1.0000	0.9997	1.0000	1.0000	0.9997		1.0000	0.9996	1.0000
PD	0.9955	0.8478	1.0000	0.9996	1.0000	1.0000	0.9997	1.0000		0.9996	1.0000
PSP	1.0000	0.9645	1.0000	1.0000	0.9456	0.9259	1.0000	0.9996	0.9996		0.9999
PiD	0.9992	0.9455	1.0000	0.9999	1.0000	1.0000	0.9999	1.0000	1.0000	0.9999	

## Abbreviations

GVD: Granulovacuolar degeneration
AD: Alzheimer's disease
LBD: Lewy body disease
AGD: Argyrophilic grain disease
CBD: Corticobasal degeneration
FTLD-U: Frontotemporal lobar degeneration
MSA: Multiple system atrophy
PD: Parkinson's disease
PiD: Pick's disease
PSP: Progressive supranuclear palsy
NFT: Neurofibrillary tangle.
